# ATF3 inhibits the tumorigenesis and progression of hepatocellular carcinoma cells via upregulation of CYR61 expression

**DOI:** 10.1186/s13046-018-0919-8

**Published:** 2018-10-30

**Authors:** Cong Chen, Chao Ge, Zheng Liu, Liangyu Li, Fangyu Zhao, Hua Tian, Taoyang Chen, Hong Li, Ming Yao, Jinjun Li

**Affiliations:** 1 State Key Laboratory of Oncogenes and Related Genes, Shanghai Cancer Institute, Renji Hospital, Shanghai Jiaotong University School of Medicine, 25/Ln 2200 Xietu Road, Shanghai, 200032 China; 2 Shanghai Medical College, Fudan University, Shanghai, China; 3 Qidong Liver Cancer Institute, Qidong, China

**Keywords:** ATF3, CYR61, Hepatocellular carcinoma

## Abstract

**Background:**

Hepatocellular carcinoma (HCC) is one of the most common malignant cancers with a high incidence and high mortality in East Asia. Identifying biomarkers and clarifying the regulatory mechanisms of HCC are of great importance. Herein, we report the role and mechanism of activating transcription factor 3 (ATF3), a member of the ATF/cAMP-responsive element-binding protein family of transcription factors in HCC.

**Methods:**

ATF3 overexpression vector and shRNAs were transfected into HCC cancer cells to upregulate or downregulate ATF3 expression. In vitro and in vivo assays were performed to investigate the functional role of ATF3 in hepatocellular carcinoma. RNA-Seq was performed to screen the differentially expressed genes downstream of ATF3. The dual-luciferase reporter assay, chromatin immunoprecipitation (Ch-IP) analysis and functional rescue experiments were used to confirm the target gene regulated by ATF3. Tissue microarrays (TMAs) comprising 236 human primary HCC tissues were obtained and immunohistochemical staining were carried out to analyze the clinical significance of ATF3.

**Results:**

The results indicate that ATF3 significantly inhibited the proliferation and mobility of HCC cells both in vitro and in vivo. Cysteine-rich angiogenic inducer 61 (CYR61) is a key target for transcriptional regulation by ATF3. Both ATF3 and CYR61 were consistently downregulated in human HCC tissues, and their expression levels were significantly and positively correlated with each other.

**Conclusions:**

Our findings indicate that ATF3 functions as a tumor suppressor in HCC through targeting and regulating CYR61.

**Electronic supplementary material:**

The online version of this article (10.1186/s13046-018-0919-8) contains supplementary material, which is available to authorized users.

## Background

Hepatocellular carcinoma (HCC), the fourth most commonly diagnosed cancer in human and one of the leading cause of cancer-related death worldwide, progresses via multiple steps accompanied by a series of progressive alterations in gene expression [1]. Great strides have been made in exploring effective diagnostic and therapeutic strategies for HCC, but there has been no significant reduction in its morbidity and mortality. According to the International Statistical Classification of Diseases and Related Health Problems, 10th revision (ICD-10), an estimated 466,100 new cases of liver cancer and 422,100 deaths related to liver cancer occurred in China in 2015 [2]. Therefore, the changes in gene expression and related genetic factors involved in HCC progression are still insufficiently understood, and there is an unmet clinical need for novel potential therapeutic targets.

Activating transcription factor 3 (ATF3), an ATF/cAMP-responsive element-binding protein (CREB) family member, was found to be involved in a broad spectrum of cellular stresses, including DNA damage [3], cellular injury [4], oxidative stress and oncogenic stimuli [5], and was also shown to regulate diverse cellular functions by either binding to the ATF/CREB cis-regulatory element or interacting with other proteins, such as p53 and NF-κB [6]. Several reports indicated that ATF3 expression is downregulated in a variety of human cancers, including colon cancer [7], liver cancer [8], multiple myeloma [9], neuroblastoma [10], bladder cancer [11], prostate cancer [12], malignant glioma [13] and non-small cell lung carcinoma [14]. ATF3 may inhibit tumor formation by inducing cell cycle arrest and apoptosis [15]. In colon cancers, ATF3 plays a role in regulating genes downstream of protein kinase-like endoplasmic reticulum kinase (PERK) and PERK-eIF2α signaling during instances of endoplasmic reticulum stress [16]; on the other hand, ATF3 plays an important role in berberine-induced apoptosis [17]. Taketani et al found that when colon cancer cells encountered DNA damage, ATF3 and p53 could synergistically act on the promoter of the DR5 gene to eventually promote TRAIL-mediated apoptosis [18]. Ri et al found that in multiple myeloma (MM), patients with higher ATF3 and ATF4 expression had longer progression-free survival (PFS) than those with lower ATF3 expression; this observation was also confirmed in chromatin immunoprecipitation (Ch-IP) experiments with MM cell lines and patient specimens [9]. ATF3 also suppresses the development of prostate cancer induced by silencing of the tumor suppressor Pten in a mouse model, and knockdown of ATF3 expression promotes the activation of the oncogenic AKT signaling pathway [19]. Although aberrant ATF3 expression is frequently found in human cancers [20], it is imperative to explore the function and mechanism of this gene, particularly in HCC.

In this study, we identified ATF3 as a tumor suppressor for inhibiting cell proliferation and metastasis in HCC. Because ATF3 is a transcription factor, we also screened and identified its downstream target genes and found that ATF3 exerted its suppressive activities through upregulating CYR61 expression. Clinically, both ATF3 and CYR61 were downregulated in HCC tissues compared with corresponding adjacent tissues, and their expression was positively correlated. ATF3 was also significantly associated with intrahepatic metastases and overall survival (OS).

## Methods

### Cell lines and cell culture

The human HCC cell line SMMC-7721 and the immortalized hepatocyte cell line L-02 were purchased from the Cell Bank of the Institute of Biochemistry and Cell Biology, Chinese Academy of Sciences (Shanghai, China). MHCC-97 L, MHCC-97H and MHCC-LM3 cells were provided by the Liver Cancer Institute of Zhongshan Hospital, Fudan University (Shanghai, China). PLC/PRF/5, SK-Hep1 and human embryonic kidney (HEK)-293 T cells were purchased from the American Type Culture Collection (Manassas, VA, USA). Huh-7 cells were obtained from the Riken Cell Bank (Tsukuba, Japan). Li-7 cells were purchased from Shanghai Sixin Biological Technology. HCC-LY5 and HCC-LY10 cell lines were established in our laboratory. All the above cell lines were cultured in Dulbecco’s modified Eagle’s medium (DMEM, Gibco, New York, USA) supplemented with 10% fetal bovine serum (FBS, Gibco) along with penicillin (100 U/mL, Sigma-Aldrich) and streptomycin (100 μg/mL, Sigma-Aldrich) at 37 °C in a humidified incubator containing 5% CO_2_. All the cells were authenticated and characterized by their respective suppliers.

### Vector construction and lentivirus infection

The coding sequences (CDS) of human *ATF3* and *CYR61* were amplified and cloned into the pWPXL lentivirus vector (Addgene, USA), pWPXL-*ATF3* and pWPXL-*CYR61* fusion expression clones were successfully obtained. shRNAs targeting *ATF3* or *CYR61* as well as a negative control (shNC) were obtained from GeneChem (Shanghai, China). The *CYR61* sequence spanning 1322 bp near the transcriptional start site (TSS) as well as its truncated and mutated variants were amplified and cloned into the pGL3 vector (Promega, Madison, WI). The target primer sequences are listed in Additional file 1: Table S1. All constructs were verified by DNA sequencing. HEK-293 T cells were transfected with these plasmids using Lipofectamine™ 2000 (Invitrogen) along with the packaging and envelope plasmids psPAX2 and pMD2.G (Addgene, USA) according to the manufacturer’s protocol. Virus particles were harvested 48 h after transfection. The HCC cells were infected with recombinant lentivirus in a 0.1% polybrene (Sigma-Aldrich) solution.

### Quantitative real-time polymerase chain reaction (qRT-PCR)

Total RNA from human primary HCC tissues and cell lines was isolated using TRIzol reagent (Invitrogen, USA) and then reverse-transcribed into cDNA using a PrimeScript™ RT Reagent Kit (TaKaRa, Japan). qRT-PCR using SYBR Premix Ex Taq (TaKaRa, Japan) was performed with an Applied Biosystems 7500 (software version 2.0.5) real-time PCR system (Thermo Scientific) in triplicate, and the values were normalized to those of the housekeeping gene *GAPDH*. The comparative CT (2^-ΔΔCT^) method was applied to analyze the qRT-PCR data. The primer sequences used to quantify the target genes are provided in Additional file 1: Table S1.

### Immunoblotting

Whole-cell or tissue extracts were lysed in RIPA buffer (Thermo Scientific, USA) containing protease inhibitor and phosphatase inhibitor (Roche, Switzerland). The resulting lysates were then electrophoresed by 12% SDS-PAGE and transferred to 0.2-μm polyvinylidene difluoride membranes (Merck Millipore). These membranes were blocked in 5% nonfat milk for 2 h at room temperature, incubated with primary antibodies at 4 °C overnight and then treated with HRP-linked secondary antibodies for 1.5 h at room temperature. After the membranes were washed with phosphate-buffered saline containing Tween 20 (PBST), the protein bands were visualized using a Pierce ECL development system (Thermo Scientific, USA) via a chemiluminescence analyzer (Bio-Rad, USA) for different exposure times. The antibody information is listed in Additional file 1: Table S2, and β-Actin (A3854, Sigma-Aldrich) was used as a loading control.

### Cell proliferation and colony formation assays

Infected cells were seeded in 96-well plates (2000 cells/well) in triplicate and cultured for 7 days to assess proliferation with the Cell Counting Kit-8 (CCK-8, bimake, USA) and the 3-[4,5-dimethylthiazol-2-yl]-2,5-diphenyltetrazolium bromide (MTT) assay. The absorbance was measured at 450 nm (CCK-8) and 570 nm (MTT). For the colony formation assay, cells were seeded in 6-well plates (1000 cells/well) and allowed to form colonies. Once colonies were visible (> 50 cells), they were fixed with 4% paraformaldehyde and stained with Giemsa (Sigma-Aldrich, USA), and the number of colonies per well was counted.

### Apoptotic assay

Annexin V-PE and 7-AAD (both available from BD Biosciences, San Jose, CA, USA) staining was used to visualize apoptotic cells according to the manufacturer’s instructions. Briefly, 1 × 10^6^ cells were seeded in 6-well plates and incubated overnight. Cells were then collected and washed twice with PBS and resuspended in 200 μl of 1× binding buffer. Next, 5 μl of the Annexin V-PE and 7-AAD solution were added, and samples were incubated for 30 min at RT and analyzed by flow cytometry.

### Migration and invasion assays

HCC cells were plated into 6-well culture plates (1 × 10^6^ cells/well) and incubated overnight. Then, the monolayer of cells was scratched to form vertical wound. After we washed the cells twice with 1× PBS and replaced the medium with DMEM containing 2% FBS and 1 mM thymidine (Sigma-Aldrich, USA), cells that migrated into the wound area were captured using an inverted fluorescence microscope (Carl ZEISS, Germany) at 100× magnification and images of the wound at 0 h, 24 h and 48 h after scratching were obtained. The migratory ability of the cells was determined by calculating the ratio of the healing width at 48 h to the wound width at 0 h.

$$ \mathrm{Relative}\ \mathrm{percentage}\ \mathrm{of}\ \mathrm{wound}\ \mathrm{healing}\ \left(\%\right)=\frac{\mathrm{the}\ \mathrm{wound}\ \mathrm{width}\ \mathrm{of}\ 0\mathrm{h}\hbox{-} \mathrm{the}\ \mathrm{wound}\ \mathrm{width}\ \mathrm{of}\ 48\mathrm{h}}{\mathrm{the}\ \mathrm{wound}\ \mathrm{width}\ \mathrm{of}\ 0\mathrm{h}}\times 100\% $$

A transwell insert with an 8-μm pore filter (Merck Millipore, USA) was precoated with 10% Matrigel (BD Bioscience, USA) and incubated for 30 min at 37 °C. A total of 1 × 10^5^ cells was seeded into the upper chamber of the insert with 200 μl of serum-free media, while the lower chamber was filled with 600 μl of complete medium as a chemoattractant. After 12 ~ 24 h of incubation, the inserts were removed, and cells in the upper chamber that did not migrate were scraped away with a cotton swab. The cells that migrated through the membrane and adhered to its lower surface were fixed with 4% paraformaldehyde for 30 min and stained with a crystal violet solution (Sigma-Aldrich, USA). Invasive cells were photographed and counted using the fluorescence microscope at 200× magnification.

### Mouse xenograft model

All treatments were performed under the guidelines of the Shanghai Medical Experimental Animal Care Commission. Forty microliters of ATF3-overexpressing or ATF3-silenced cells in serum-free DMEM/Matrigel (1:1, *v*/v) at a final concentration of 5 × 10^7^ cells/ml were orthotopically inoculated into the left hepatic lobes of 6~ 8-week-old male mice using a microsyringe. Mice were sacrificed at 6~ 8 weeks after injection. Then, the tumor xenografts within the liver were weighed, and the liver and lung tissues were fixed in 4% buffered formalin and subjected to a routine preparation of paraffin embedding, sectioning, hematoxylin-eosin (H&E) staining and immunohistochemical staining.

### RNA-seq

ATF3-overexpressing SK-Hep1 cells and ATF3-silenced SMMC-7721 cells were used for RNA-seq analysis by Shanghai Biotechnology Corporation (Shanghai, China) in duplicate. Part of the differentially expressed genes were available in Additional file 1: Table S3–S4, and the whole raw sequencing data of this study have been deposited at DNA Data Bank of Japan (DDBJ) under the accession number DRA007320 and is available for download at: https://www.ddbj.nig.ac.jp/index-e.html.

### Dual-luciferase reporter assays

Cells were plated in 48-well culture plates to allow adhesion, followed by transient cotransfection with pWPXL or *ATF3* plasmids, *CYR61* promoters, and the PRL-TK reporter construct using Lipofectamine™ 2000 (Invitrogen). After 48 h, the *Renilla* and firefly luciferase activities were determined according to the manufacturer’s instructions (Promega).

### Ch-IP

The Ch-IP assay was performed in 293 T, SMMC-7721 and Huh-7 cells. The cells were cross-linked with 10% formaldehyde and then quenched with 1 M glycine. After the cells were washed with 1× PBS, they were incubated in Tissue Protein Extraction Reagent (Thermo Scientific) for 5 min in an ice bath and centrifuged at 2000 rpm for 5 min. The sediments were suspended in nuclear lysis buffer, and DNA was sheared into fragments of 200~ 500 bp by sonication. The nuclear lysate was incubated with specific antibody and protein A/G agarose beads (Sigma-Aldrich) at 4 °C overnight on a rotator. After reversing the crosslinks, the DNA was isolated and used for PCR analysis with the primers listed in Additional file 1: Table S1.

### Immunohistochemical analysis

Tissue microarrays (TMAs) comprising 236 human primary HCC tissues obtained from the Qidong Liver Cancer Institute were constructed, and staining was performed as previously described [21]. The samples were photographed using a Leica SCN400 slide scanner (Meyer Instruments, Houston, TX, USA) and analyzed by semiquantitative scoring. Immunohistochemical scores were obtained as follows: the intensity of staining was categorized as 0 or 1 for low or high protein expression, respectively. The antibodies used are listed in Additional file 1: Table S5.

### Statistical analysis

Values are presented as the mean ± standard deviation (S.D.) with at least three independent experiments. The data were analyzed using SPSS version 16.0 (SPSS, Inc., Chicago, IL, USA). Data analysis was conducted by paired or unpaired two-tailed Student’s *t*-tests. The relationship between ATF3 and CYR61 expression was analyzed using Spearman’s correlation coefficient. Survival analysis was performed using the Kaplan–Meier method. Survival prognosis was investigated with univariate Cox regression models followed by a multivariate Cox regression model. Statistical significance was defined as *P < 0.05 and **P < 0.01.

## Results

### ATF3 inhibited HCC cell proliferation in vitro and HCC cell tumorigenesis in vivo

**Fig. 1:**
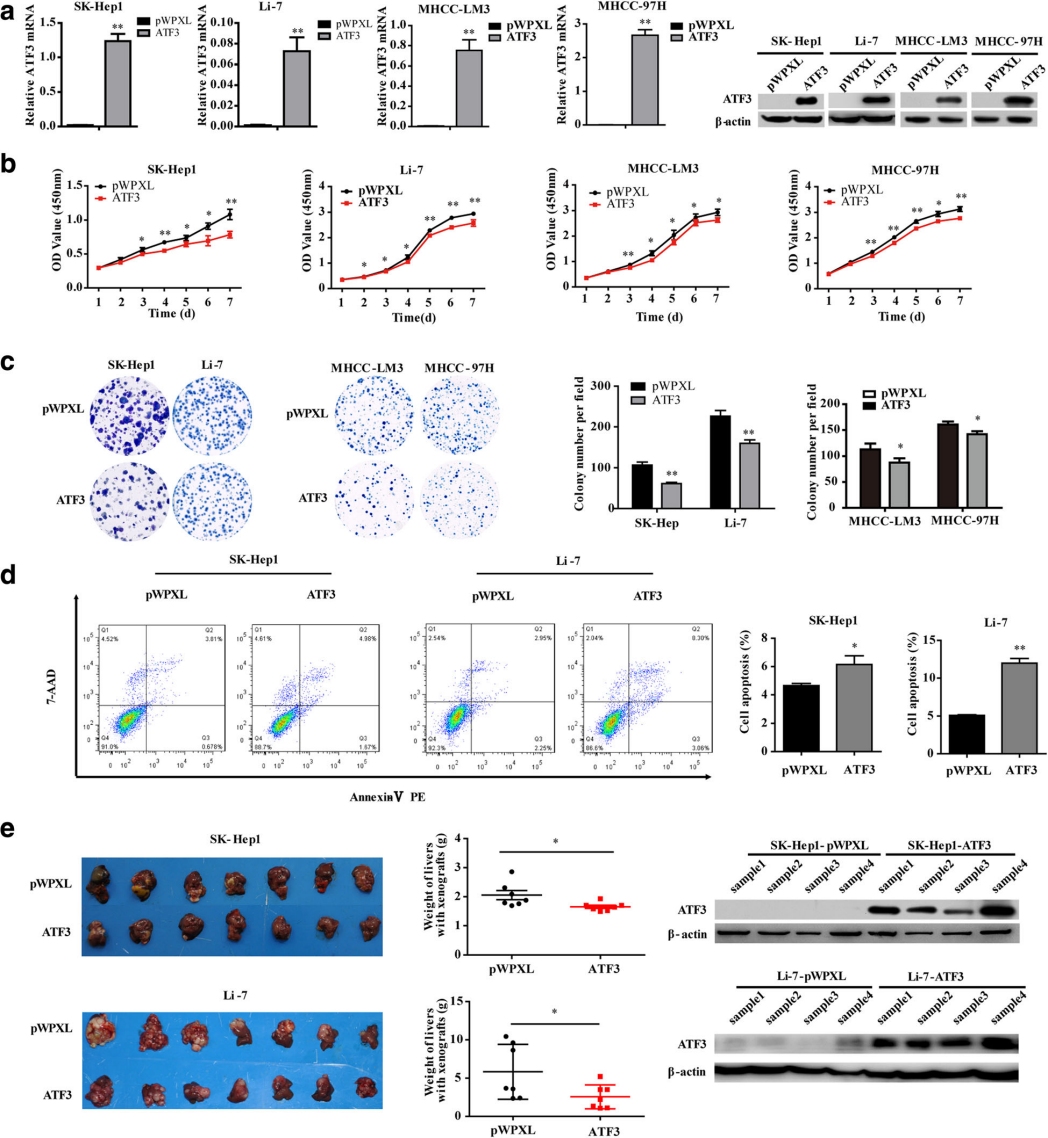
ATF3 inhibited HCC cell proliferation in vitro and tumorigenesis in vivo. **a** As a quality control, in vitro ATF3 mRNA and protein expression was detected by qRT-PCR and western blot, respectively, in SK-Hep1, Li-7, MHCC-LM3 and MHCC-97H cells with ectopic ATF3 expression via the lentiviral vector (pWPXL). **b** The cell proliferative ability of HCC cells with ATF3 overexpression was inhibited as indicated by the CCK-8 assay. **c** The colony forming ability of HCC cells with ATF3 overexpression was reduced. **d** The percent of cell apoptosis with ATF3 overexpression in SK-Hep1 and Li-7 was increased. **e** Images of liver tissues collected from nude mice with tumor xenografts derived from SK-Hep1 (left top panel) and Li-7 (left bottom panel) cells with stable overexpression of ATF3. The livers with xenografts were weighed and compared (middle top and bottom panel). ATF3 protein expression in tissue samples of the xenografts was detected by western blot (right top and bottom panel). β-Actin was used as a loading control. The bar graphs in (**a**), (**b**), (**c**) and (**d**) represent the quantitative data from three independent experiments. Unpaired Student’s *t*-test was used for statistical analysis, and the data are shown as the mean ± S.D. *P < 0.05 and **P < 0.01

To investigate the biological effects of ATF3 in HCC cells, the mRNA and protein levels of ATF3 in HCC cell lines and the immortalized human hepatocyte cell line L-02 were analyzed by qRT-PCR and western blot, respectively (Additional file 1: Figure S1). We found that the SK-Hep1, Li-7, MHCC-LM3 and MHCC-97H cell lines had lower endogenous expression levels of ATF3, and these four HCC cell lines were used to establish cells stably overexpressing ATF3 (Fig. 1a), which were used in the following functional experiments. Cells infected with empty pWPXL vector served as a control group. The CCK-8, MTT assays and in vitro colony formation assay for cell viability indicated that ATF3 overexpression inhibited HCC cell proliferation in vitro (Fig. 1b, c and Additional file 1: Figure S2a). The analysis of flow cytometry for cell apoptosis indicated that ATF3 overexpression increased the percent of cell apoptosis (Fig. 1d).

We sought to determine whether ATF3 could exert an inhibitory effect on tumorigenesis in vivo. The SK-Hep1 and Li-7 cells with stable ATF3 overexpression were orthotopically injected into the livers of immunodeficient nude mice, and the mice injected with the corresponding empty pWPXL vector served as a control group. The results indicated that the average weights of livers with tumor xenografts were significantly lower in mice injected with cells overexpressing ATF3. Furthermore, the tissues of the xenografts overexpressing ATF3 still maintained elevated ATF3 expression levels (Fig. 1e). These results suggested that ATF3 could repress cell proliferation in vivo, which was consistent with the observed in vitro results.

### Silencing ATF3 promotes tumor cell proliferation in vitro and in vivo

**Fig. 2:**
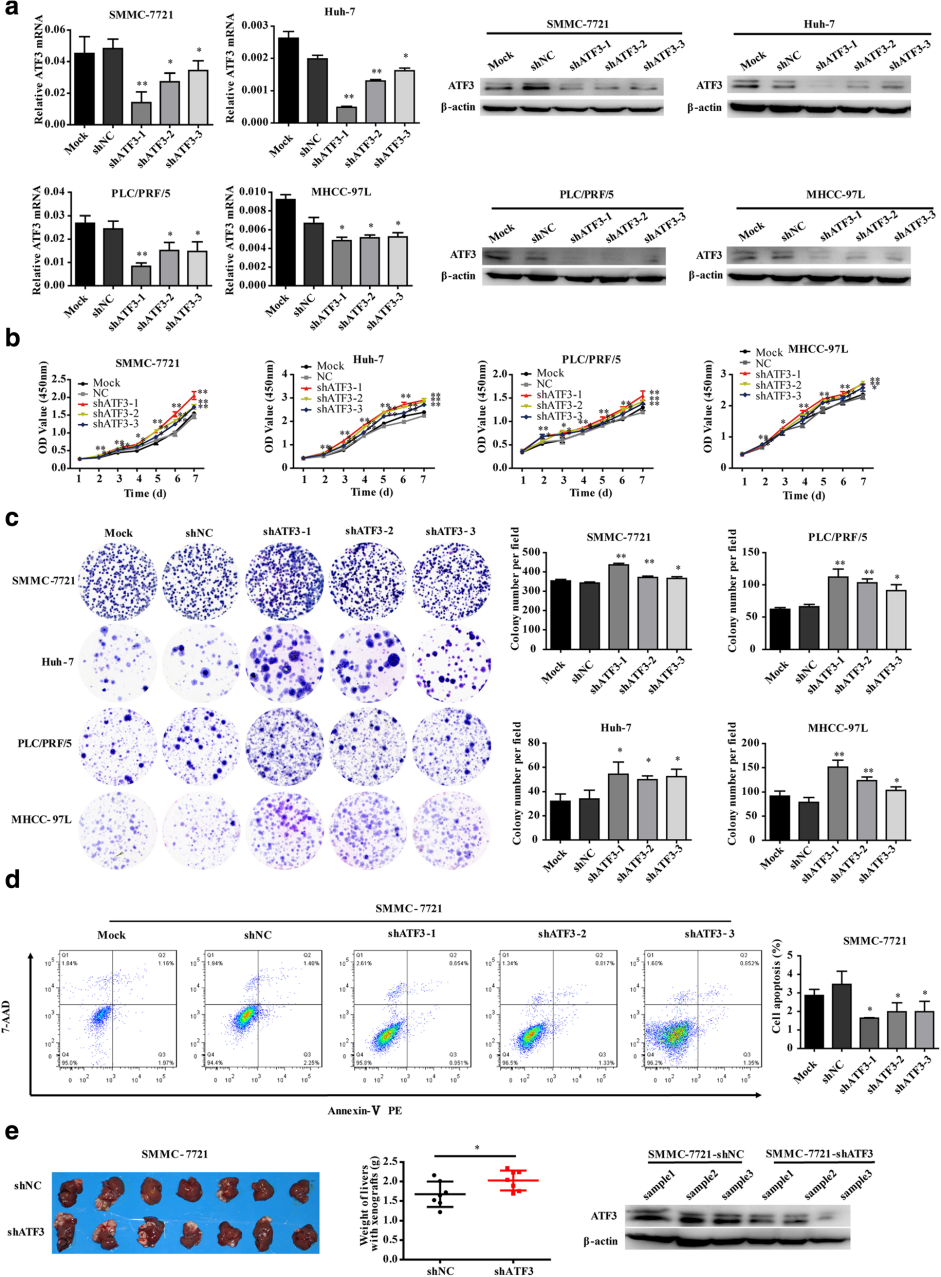
Silencing ATF3 promoted tumor cell proliferation in vitro and in vivo. **a** As a quality control, in vitro ATF3 mRNA and protein expression was detected by qRT-PCR and western blot, respectively, in SMMC-7721, Huh-7, PLC/PRF/5 and MHCC-97 L cells with stable knockdown of ATF3. Mock and shNC-expressing cells served as blank and negative control groups, respectively. Compared to the NC and mock groups, SMMC-7721, Huh-7, PLC/PRF/5 and MHCC-97 L cells expressing shATF3 showed increased cell proliferation (**b**) and colony forming ability (**c**). **d** The percent of cell apoptosis with ATF3 knockdown in SMMC-7721 was decreased. **e** Images of liver tissues collected from nude mice with tumor xenografts derived from SMMC-7721 (left panel) cells with stable knockdown of ATF3. The livers with xenografts were weighed (middle panel). The ATF3 protein level in tissue samples from the xenografts was detected by western blot (right panel). β-Actin was used as a loading control. The bar graphs in (**a**), (**b**), (**c**) and (**d**) represent the quantitative data from three independent experiments. Unpaired Student’s *t*-test was used for statistical analysis, and the data are shown as the mean ± S.D. *P < 0.05 and **P < 0.01

Given that ATF3 is associated with cell proliferation in vitro and in vivo, we examined whether knocking down ATF3 in HCC cells would enhance their proliferative abilities. As previously observed, ATF3 was differentially expressed in the panel of HCC cell lines, and three lentiviral vectors expressing effective shRNAs targeting ATF3 were used to knockdown endogenous ATF3 expression in the four cell lines with high ATF3 expression (Fig. 2a). The results showed that ATF3 knockdown in SMMC-7721, Huh-7, PLC/PRF/5 and MHCC-97 L cells showed markedly enhanced proliferative and colony forming abilities (Fig. 2b, c and Additional file 1: Figure S2b), and decreased cell apoptosis (Fig. 2d).

We next examined whether silencing ATF3 could enhance tumorigenesis in vivo. SMMC-7721 cells with stable ATF3 knockdown were orthotopically transplanted into the livers of immunodeficient nude mice, with mice injected with cells expressing the corresponding scrambled shRNA (shNC) vector serving as a control group. The results indicated that the average weights of livers with tumor xenografts were significantly higher in mice injected with ATF3-silenced cells. Meanwhile, the tissues of xenografts derived from cells with ATF3 knockdown still maintained the low levels of ATF3 expression (Fig. 2e). These results suggested that knocking down ATF3 could reverse the effects elicited by ATF3 overexpression in vitro and in vivo.

### ATF3 suppressed HCC cell migration and invasion in vitro and metastasis in vivo

**Fig. 3:**
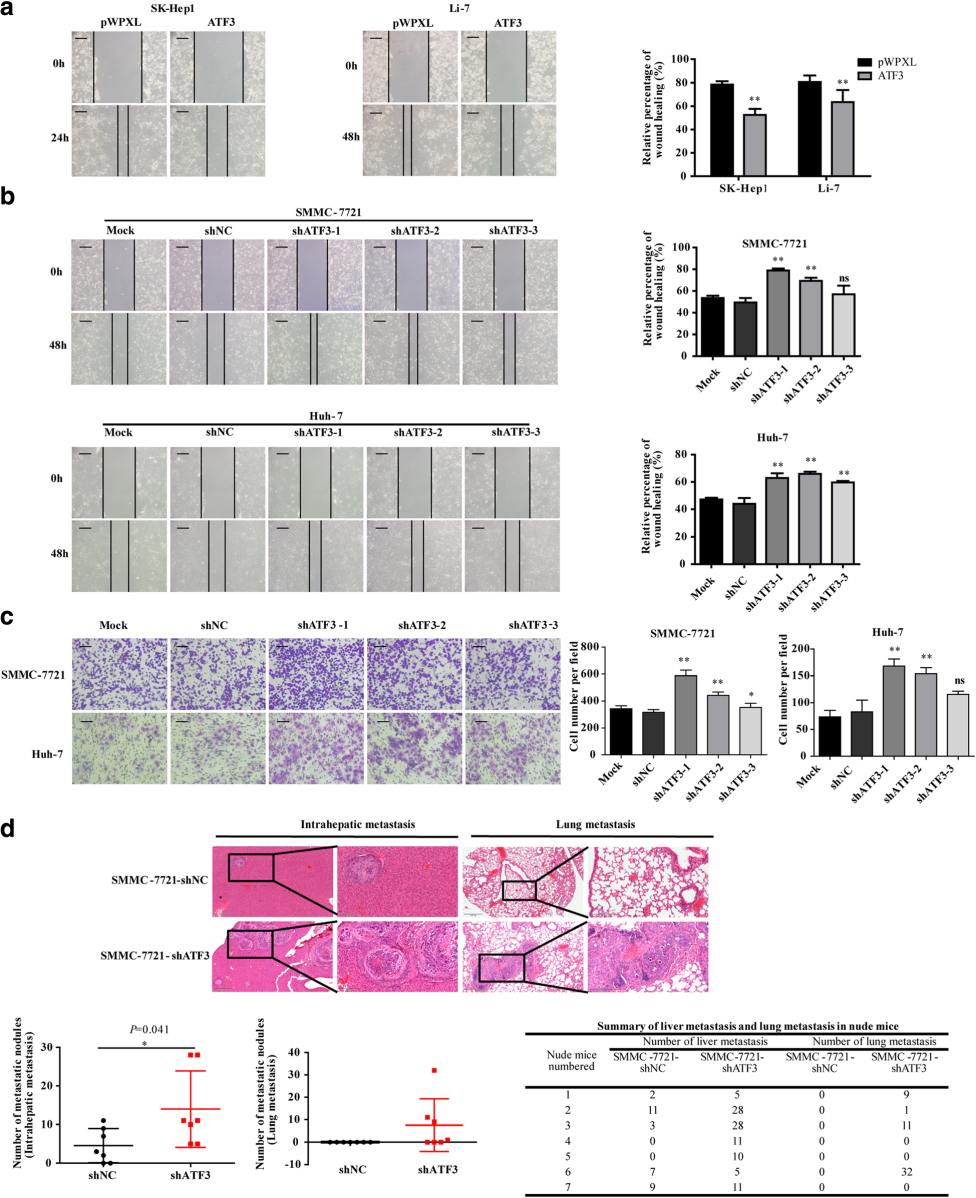
ATF3 suppressed HCC cell mobility in vitro and metastasis in vivo. **a** Overexpression of ATF3 suppressed HCC cell migration in vitro as assessed by the wound healing assay (scale bar, 200 μm). **b** Knockdown of ATF3 (shATF3–1, − 2, − 3) promoted HCC cell migration in vitro as assessed by the wound healing assay (scale bar, 200 μm). **c** Silencing ATF3 promoted HCC cell invasion in vitro (scale bar, 100 μm). The bar graphs in (**a**), (**b**) and (**c**) represent quantitative data from three replicates. **d** Representative images show the intrahepatic metastatic and lung metastatic nodules derived from SMMC-7721 cells with silenced ATF3 and those derived from the control cells (left images, scale bar, 500 μm; right images, scale bar, 100 μm). The numbers of intrahepatic metastatic and lung metastatic nodules are presented in the bottom panel (*n* = 7). Unpaired Student’s *t*-test was used for statistical analysis, and the data are shown as the mean ± S.D. *P < 0.05, **P < 0.01 and ns: no significance

To explore the migratory and invasive abilities of ATF3 in HCC cells, we performed the wound healing and transwell invasion assay. The results revealed that ATF3 overexpression in SK-Hep1, Li-7, MHCC-LM3 and MHCC-97H cells decreased the migratory and invasive abilities compared to their respective pWPXL-transduced cell groups (Fig. 3a and Additional file 1: Figure S3a and b). However, knockdown of ATF3 in SMMC-7721, Huh-7, PLC/PRF/5 and MHCC-97 L cells increased the migratory and invasive abilities compared to those in the corresponding shNC groups (Fig. 3b, c and Additional file 1: Figure 3c and d).

To observe the effect of ATF3 on tumor metastasis, histological examination of formalin-fixed paraffin-embedded liver and lung tissue sections from mice orthotopically injected with SMMC-7721-shRNA cells were carried out. The results showed that sections from the SMMC-7721-shRNA group had more intrahepatic and lung metastatic nodules than those from the control mice (*P* = 0.041, Fig. 3d). These results indicated that ATF3 markedly inhibited the in vitro mobility and the in vivo metastasis of HCC cells.

### CYR61 is a target gene of ATF3

**Fig. 4:**
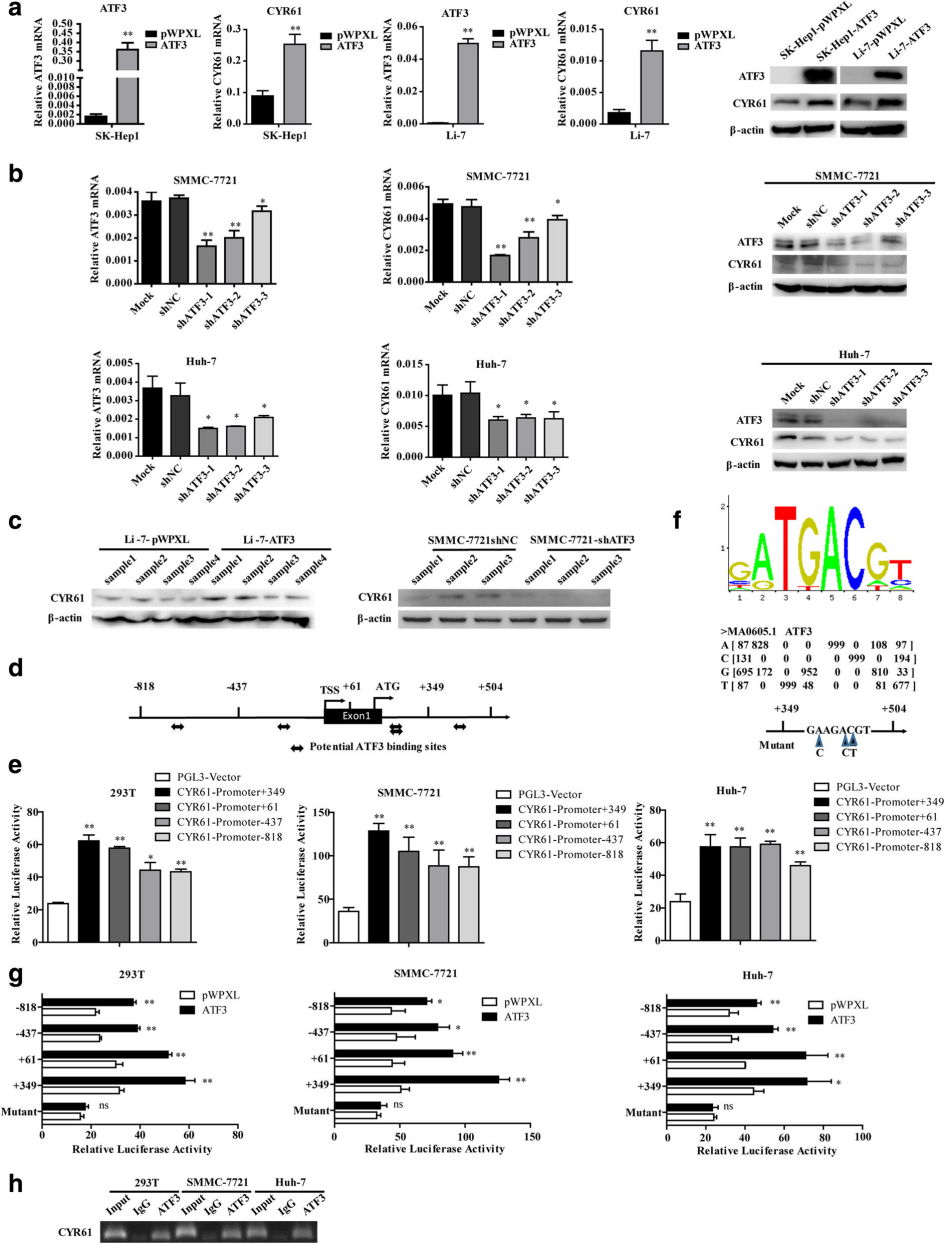
ATF3 upregulated CYR61 by directly binding to its consensus binding sequence on the *CYR61* gene*.* **a** qRT-PCR and western blot showed that SK-Hep1 and Li-7 cells overexpressing ATF3 had increased CYR61 mRNA and protein expression. **b** qRT-PCR and western blot showed that SMMC-7721 and Huh-7 cells with ATF3 knockdown had decreased CYR61 mRNA and protein expression. **c** Upregulated expression of CYR61 protein in tissue samples from xenografts was detected by western blot. **d** Potential ATF3 binding sites next to the transcriptional start site of the *CYR61* sequence were identified with the JASPAR database (http://jaspar.genereg.net/). **e** The relative luciferase activities of the full CYR61 sequence and the truncated construct in 293 T, SMMC-7721 and Huh-7 cells. **f** The sequence logo of a potential ATF3 binding site in JASPAR and a diagram of mutant sites in the CYR61 sequence. **g** The relative luciferase activities of the truncated and mutant constructs of the CYR61 sequence in 293 T, SMMC-7721 and Huh-7 cells transfected with either ATF3 or pWPXL. **h** Assessment of ATF3 binding to the CYR61 sequence in 293 T, SMMC-7721 and Huh-7 cells was performed by Ch-IP using an antibody against ATF3 and a negative control (IgG). Agarose gel electrophoresis was used to analyze the crosslinking status. Data are shown as the mean ± S.D. from experiments with three replicates. *P < 0.05, **P < 0.01 and ns: no significance

ATF3, as a transcription factor, its direct targets that have been purported to be responsible for its functional effects were investigated. The differentially expressed genes were selected from the RNA-Seq data from SK-Hep1-pWPXL/ATF3 and SMMC-7721-shNC/shATF3 cells (Additional file 1: Tables S3 and S4), and *CYR61* was noted and used for subsequent experiments because it was unanimously positively correlated with ATF3 expression at the mRNA and protein levels both in the ATF3-overexpressing and ATF3-silenced cells as well as in primary tissues (Fig. 4a–c and Additional file 1: Figure S5).

To further identify whether *ATF3* could regulate *CYR61*, the JASPAR (jaspar.genereg.net) database was used to analyze the potential *ATF3* binding site in the *CYR61* sequence (Fig. 4d), which spans 1322 bp (− 818~ + 504) near the TSS. This segment was cloned and inserted into the pGL3 vector. We also deleted the sequences that span the region from − 437, + 61 and + 349 to + 504 (fixed + 504 as the universal R), these truncated sequences all showed higher luciferase activity than the pGL3 vector in 293 T, SMMC-7721 and Huh-7 cells (Fig. 4e). According to the sequence logo of the potential ATF3 binding site determined by JASPAR (Fig. 4f), we mutated the predicted ATF3 binding site (ATGACGT) on CYR61 sequence (+ 349 to + 504 bp) and found that the relative luciferase activity of these truncated sequences was significantly increased when ATF3 was overexpressed but the enhanced luciferase activity was reversed by transfection of the mutant (Fig. 4g). The result of the Ch-IP assay further verified that *ATF3* tightly binds to this transcriptional area of the *CYR61* gene (Fig. 4h). Taken together, these data suggest that *CYR61* is a direct transcriptional target of *ATF3*.

### CYR61 suppressed HCC cell proliferation and migration in vitro

**Fig. 5:**
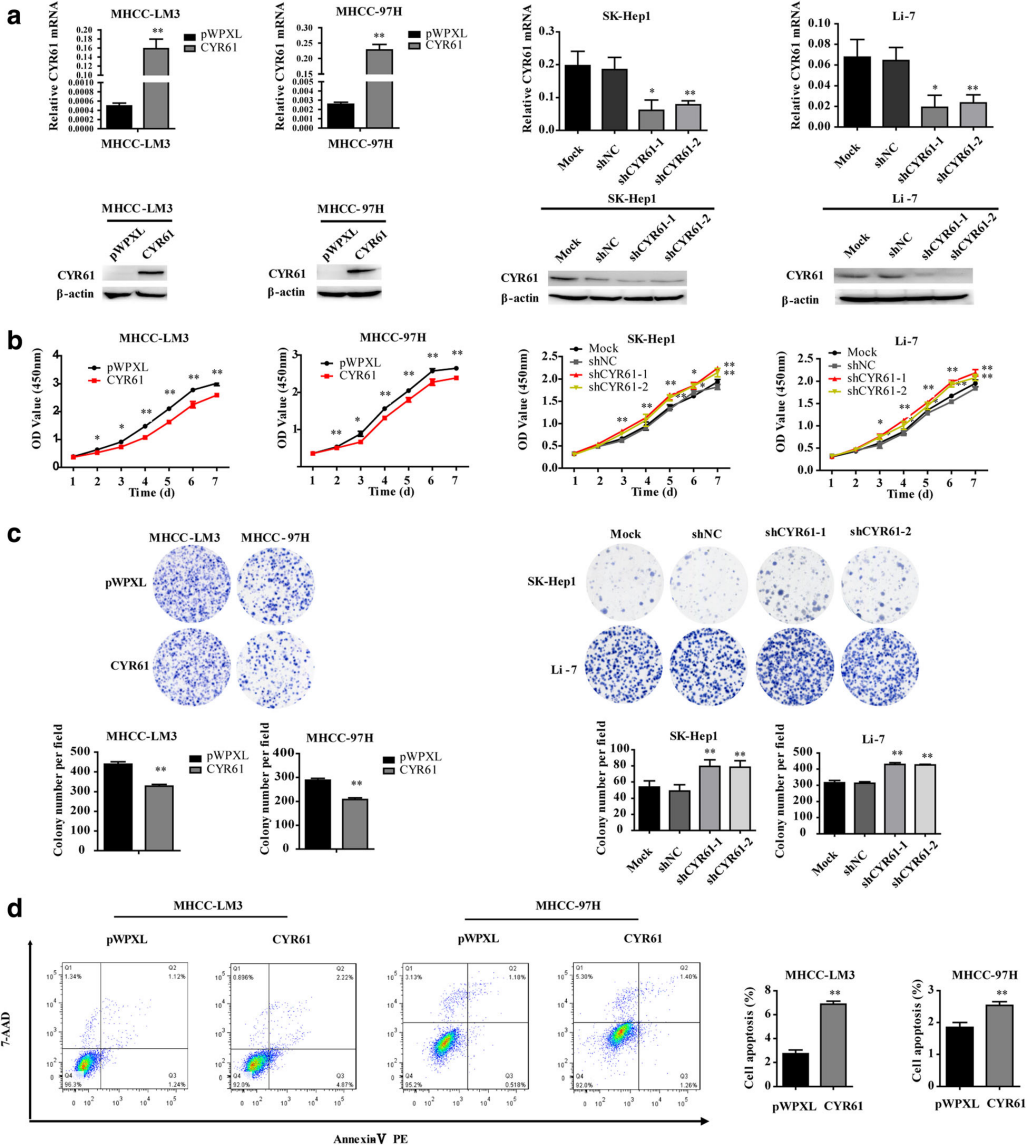
CYR61 suppressed HCC cell proliferation in vitro. **a** As a quality control, in vitro CYR61 mRNA and protein expression was detected by qRT-PCR and western blot, respectively, in MHCC-LM3 and MHCC-97H cells with ectopic CYR61 expression via the lentiviral vector (pWPXL) and SK-Hep1, Li-7 cells with silencing of CYR61. The cell proliferative ability of HCC cells with CYR61 overexpression and knockdown were examined by the CCK-8 assay (**b**) and the colony formation assay (**c**). **d** The percent of cell apoptosis with CYR61 overexpression was increased. The bar graphs in (**a**), (**b**), (**c**) and (**d**) represent the quantitative data from three independent experiments. Unpaired Student’s t-test was used for statistical analysis, and the data are shown as the mean ± S.D. *P < 0.05 and **P < 0.01

To determine the biological function of CYR61 in HCC cell lines, we selected MHCC-LM3 and MHCC-97H to establish stably overexpressing CYR61 cell lines, SK-Hep1 and Li-7 to establish stably silencing CYR61 cell lines (Fig. 5a). The results revealed that CYR61 overexpression decreased the proliferative and migratory abilities compared to their respective pWPXL-transduced cell groups. On the contrary, CYR61 knockdown increased the proliferative and migratory abilities compared to shNC group (Fig. 5b, c and Additional file 1: Figure S4). To further investigate the effect of CYR61 on apoptosis, flow cytometry indicated that CYR61 overexpression could increase the apoptosis in MHCC-LM3 and MHCC-97H cells (Fig. 5d).

### CYR61 is involved in the suppressive effect of ATF3 on HCC cells

**Fig. 6:**
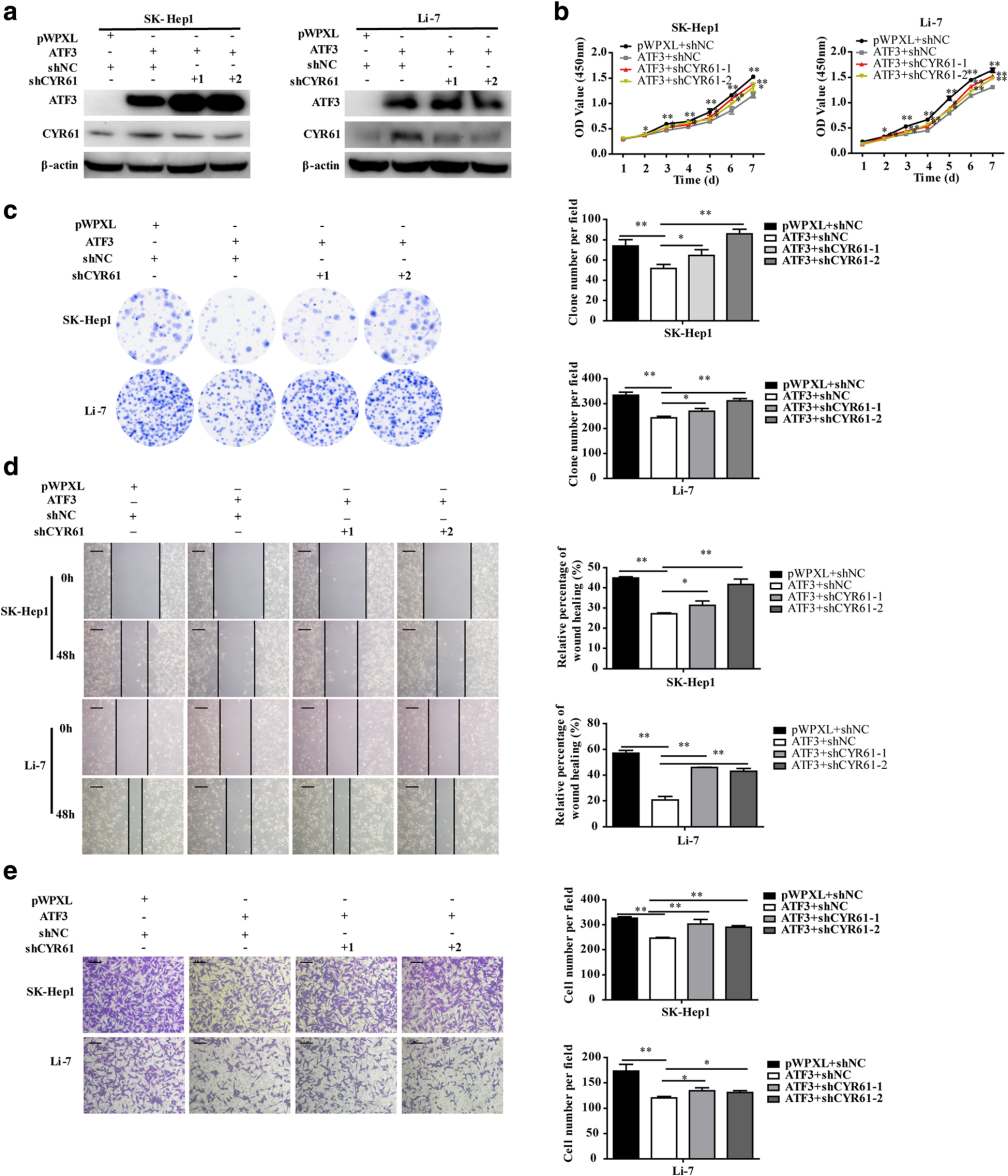
Knockdown CYR61 in HCC cells overexpressing ATF3 rescued the suppressive effects of ATF3 in vitro. **a** Western blot of CYR61 protein expression in ATF3-overexpressing HCC cells with CYR61 knockdown (shCYR61–1, − 2). β-Actin was used as a loading control. The influence of CYR61 knockdown on the inhibitory effects of ATF3 on cell growth (**b**), colony formation (**d**), migration (**d**) and invasion (**e**) in vitro were detected by the CCK-8 assay, colony formation assay, wound healing assay (scale bar, 200 μm) and transwell invasion assay (scale bar, 100 μm), respectively. The bar graphs in (**b**), (**c**), (**d**) and (**e**) represent quantitative data from three replicates. Data are shown as the mean ± S.D. from experiments with three replicates. *P < 0.05 and **P < 0.01

To confirm that CYR61 was functional downstream of ATF3, functional rescue experiments were performed. CYR61 expression was knocked down (shCYR61–1 and shCYR61–2) in SK-Hep1 and Li-7 cells with stable overexpression of ATF3 (Fig. 6a). In vitro evaluation of these cells by the CCK-8 and MTT and colony formation assays revealed that knockdown of CYR61 significantly reversed ATF3-induced inhibition of cell proliferation (Fig. 6b, c and Additional file 1: Figure S2c), and the inhibited migratory and invasive abilities of ATF3 were also reversed after CYR61 interference (Fig. 6d and e). Our above findings demonstrated that *CYR61* is a functional target of *ATF3* and is responsible for the suppressive effects of ATF3 in HCC cells.

### Differential expression of ATF3 and CYR61, correlations between ATF3 and CYR61 expression in HCC tissues, and the clinical significance of these findings

**Fig. 7:**
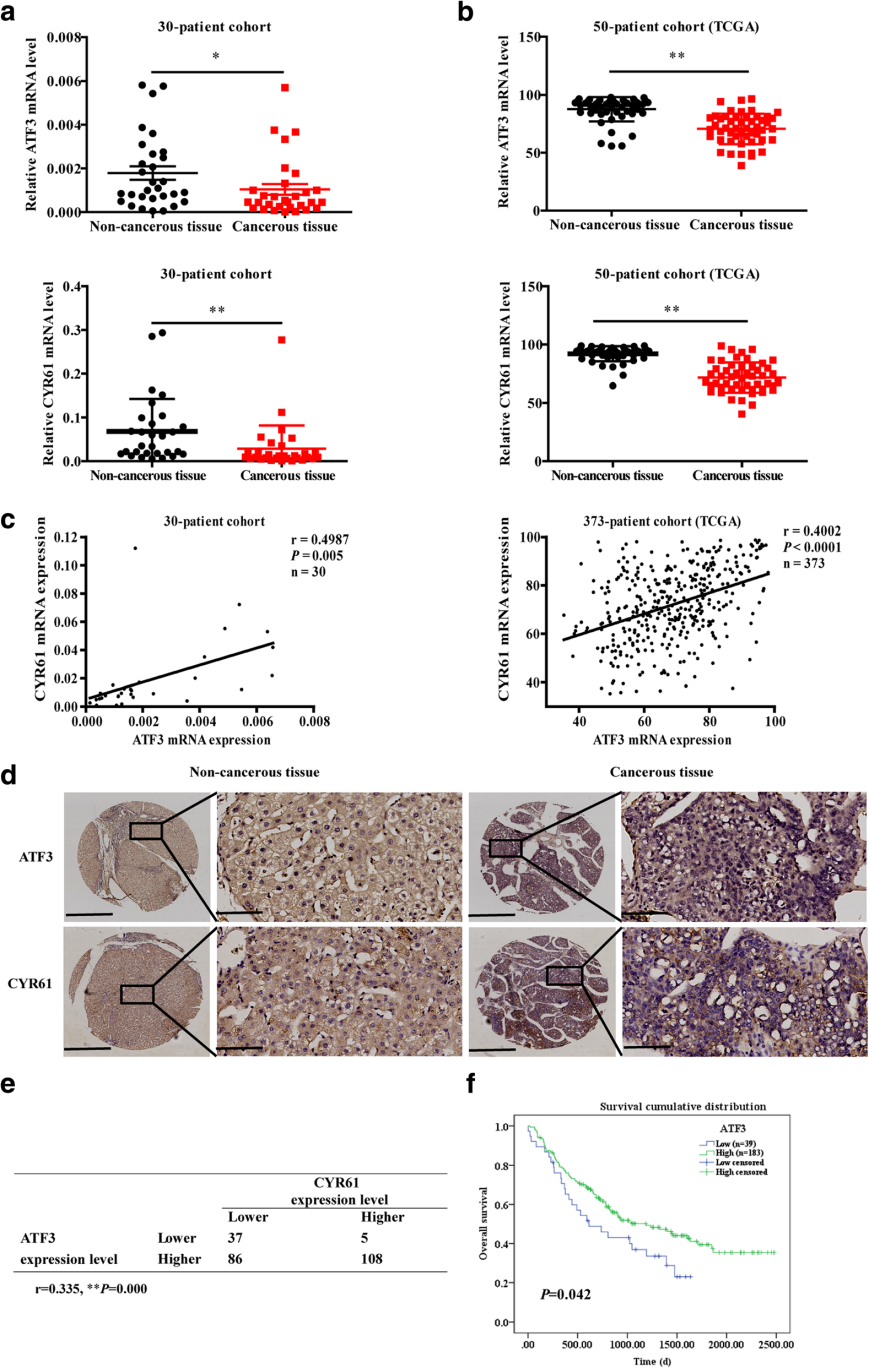
ATF3 expression was positively correlated with CYR61 expression in primary HCC tissues. The mRNA expression of ATF3 and CYR61 in 30 pairs of HCC and noncancerous liver tissues was detected by qRT-PCR (**a**), which was also used to analyze 50 pairs of cancerous and noncancerous liver tissues from the TCGA (**b**). **c** The correlation between ATF3 and CYR61 mRNA levels in 30 paired cancerous/noncancerous liver tissues from primary HCC patients and 373 paired samples from the TCGA cohort. The Pearson correlation coefficients (r) and *p* value are indicated. **d** Representative images of ATF3 and CYR61 protein staining by IHC in cancerous /noncancerous liver tissues (scale bar, 100 μm.). **e** The positive correlation between ATF3 and CYR61 protein levels in 236 human primary HCC tissues is presented. r, Pearson correlation coefficient; *p, p* value. **f** Kaplan–Meier analysis of overall survival in 222 HCC patients, as stratified by ATF3 expression. The log-rank test was used to determine statistical significance. *P < 0.05 and **P < 0.01

To further investigate the expression and correlation of ATF3 and CYR61, we performed qRT-PCR to detect the general mRNA expression levels of ATF3 and CYR61 in 30 pairs of human primary HCC tissues and matched adjacent noncancerous liver tissues. The results showed that the mRNA expression of both genes was significantly downregulated in HCC tissues compared to the levels in the noncancerous liver tissues (Fig. 7a); these data were coincident with those of The Cancer Genome Atlas (TCGA) (Fig. 7b). Of note, CYR61 mRNA expression was also positively associated with ATF3 expression in HCC tissues (Fig. 7c). Immunohistochemical staining of ATF3 and CYR61 was carried out, and the expression intensities of ATF3 and CYR61 were scored as 0 and 1 for weak and strong immunostaining, respectively. Out of the 236 cases, 194 (82.2%) had higher ATF3 protein expression in HCC tissues while 236 (100%) adjacent non-cancerous liver tissues had higher ATF3 protein expression (*P* = 0.000), 113 (47.88%) had higher CYR61 protein expression in HCC tissues while 204 (86.44%) adjacent non-cancerous liver tissues had higher CYR61 protein expression (*P* = 0.000). The results also indicated a positive relationship in expression between the two proteins (*P* = 0.000, Fig. 7d and e). Regarding the clinicopathological correlation, lower ATF3 expression was significantly associated with the incidence of intrahepatic metastases in HCC patients (*P* = 0.039, Table 1). Furthermore, a Kaplan–Meier survival analysis revealed that patients whose primary HCC samples with higher ATF3 expression had longer OS than patients with lower ATF3 expression (*P* = 0.042; Fig. 7f). These results suggest that the protein expression levels of ATF3 and CYR61 are positively correlated in human primary HCC tissues and could be potential prognostic indicators for HCC patients.

**Table 1:** Relationship between ATF3 protein expression and clinicopathological features in HCC patients

Clinicopathological Features	Number of cases	ATF3 expression		*P* Value
		Low N (%)	High N (%)	
Age (years)				
≤ 50	159	27 (64.30)	132 (68.40)	0.606
> 50	76	15 (35.70)	61 (31.60)	
Gender				
Male	190	34 (81.00)	156 (80.40)	0.936
Female	46	8 (19.00)	38 (19.60)	
Tumor size				
≤ 5 cm	113	19 (47.50)	94 (49.70)	0.797
> 5 cm	116	21 (52.50)	95 (50.30)	
AFP (ng/ml)				
≤ 20	79	12 (28.60)	67 (35.30)	0.408
> 20	153	30 (71.40)	123 (64.70)	
HBV infection				
Negative	42	9 (22.50)	33 (17.50)	0.454
Positive	187	31 (77.50)	156 (82.50)	
Cirrhosis				
Absent	38	10 (23.80)	28 (14.40)	0.134
Present	137	32 (76.20)	166 (85.60)	
Intrahepatic metastasis				
Absent	161	23 (54.80)	138 (71.10)	0.039*
Present	75	19 (45.20)	56 (28.90)	
Histological grade				
I, II		19 (45.2)	100 (51.5)	0.458
III, IV		23 (54.8)	94 (48.5)	

*AFP* alpha-fetoprotein, *N* Number of cases, *P* value represents the probability from a Chi-square test for different immunohistochemical scores of ATF3 in HCC tissues. *P < 0.05

## Discussion

ATF3, a transcriptional repressor in the ATF/CREB family, contains only one functional domain (the basic leucine zipper domain, bZIP) that binds to the ATF/CREB cis-regulatory element (5’-TGACGTCA-3′) while dimerizing with other ATF/CREB proteins [22]. Normally, ATF3 is rarely detectable under basal conditions in most cells, but its expression can be immediately induced upon exposure of cells to a broad spectrum of stimuli, such as anticancer drugs, toxic chemicals, endoplasmic reticulum stress, DNA damage, proteasome inhibitors, oxidative stress, oncogenic stimuli, genotoxic agents, homocysteine and ischemia-reperfusion, all of which can induce cell cycle arrest and apoptosis [23, 24].

ATF3 participates in a number of cellular signal transduction pathways that include proteins such as p53 [25], TGF-β, NF-κB, Toll-like receptor 4 [26] and mouse double minute 2 (MDM2) by either interacting with other proteins or binding to the consensus ATF/CREB cis-regulatory element [5]. Previous studies demonstrated that in response to DNA damage, ATF3 could activate the tumor suppressor p53 and regulate the expression of target genes downstream of p53 [27].

ATF3 may play different roles in various tumors. Li et al. [28] found that ATF3 was reduced in esophageal squamous cell carcinoma (ESCC) compared with non-tumor adjacent tissues and ATF3 suppressed ESCC via downregulation of ID1. In bladder cancer, ATF3 suppresses metastasis of bladder cancer by regulating gelsolin-mediated remodeling of the actin cytoskeleton [11]. Reactivation of ATF3 by pracinostat is a determining factor in the tumor response to the HDACi therapy and ATF3 was a biomarker of tumor response [29]. On the other hand, upregulation of ATF3 in lung cancer could promote cell proliferation, migration, and invasion [30]. ATF3 plays contradictory functions in different diseases may be due to the complex tumor microenvironment, such as the community of genomically altered cancer cells, non-neoplastic cells, and a diverse collection of microorganisms, as well as complex crosstalk of intracellular molecules and multi-module activated signal transduction pathways. In hepatocellular carcinoma, ATF3 expression is lower in patients with advanced HCC and capsule invasion [8]. Consistent with this result, our clinical pathological analysis indicated that ATF3 was relatively downregulated in cancerous tissues compared with corresponding noncancerous liver tissues. However, we observed that ATF3 was negatively correlated with intrahepatic metastasis and was positively associated with the OS of HCC patients (Fig. 7). Our experiments further confirmed that ATF3 could suppress HCC cell proliferation and metastasis both in vitro and in vivo.

Because of ATF3’s role as a transcription factor, identifying the target genes of ATF3 is needed to better understand its physiological significance in HCC. Based on the results of the RNA-Seq analysis in HCC cells with overexpressed or silenced ATF3, we found that the most obviously affected gene is *CYR61*, which is a member of the CCN family that acts as an immediate-early gene in fibroblasts following exposure to growth stimuli [31]. CYR61 expression could be induced during hepatic injuries, and the protein functions to restrict and resolve liver fibrosis [32]. Recent studies have shown that CYR61 exhibits a protective role in wound healing and tissue repair [33]. Some reports have stated that CYR61 mediates diverse functions, including extracellular matrix formation, differentiation, cell proliferation, adhesion, migration, survival, angiogenesis and tumorigenesis [34–36], and acts as a tumor suppressor in hepatocarcinogenesis via p53 and the DNA damage response [37]. Chen et al considered that CYR61 may suppress HCC through both apoptotic and growth inhibitory mechanisms to prevent tumor progression during the very early stages of oncogenesis; one such example is suppression of hepatocarcinogenesis by inhibiting compensatory EGFR-dependent hepatocyte proliferation through integrin α6-ROS-p38 MAPK-mediated activation of p53 [38]. On the contrary, there were still some other reports showed that CYR61 could lead to the carcinogenesis in the HCC [39, 40]. In non-small cell lung cancer, CYR61 overexpression remarkably inhibited the proliferation of cancer cells by arresting them in G1 phase, prominently upregulated the expression of p53 and p21 (WAF1), and decreased the activity of cyclin-dependent kinase 2 [41]. The increased apoptosis via Cyr61-induced caspase-3 activation and depolarization of the mitochondrial membrane suppressed the growth of endometrial cancer cells [42]. Juric et al found that CYR61 is a physiologic regulator of Fas-mediated apoptosis and that the extracellular matrix microenvironment can modulate Fas-dependent apoptosis through CYR61 expression [43]. Consistent with these reports, our experiment showed that CYR61 could induce apoptosis and decrease the proliferation of HCC cells. On the other hand, the rescue experiments confirmed that CYR61 knockdown could remarkably decrease the anti-cancer function of ATF3 overexpression.

## Conclusions

To the best of our knowledge, this is the first study to explore the relationship between ATF3 and CYR61 in HCC, and our results indicate that ATF3 up-regulates the expression of CYR61 through directly binding to an area near the TSS of *CYR61*, which contributes to the inhibition of HCC progression.

## **Additional file**

Additional file 1: **Table S1.** Sequences of primers, probes and shRNA used for experiments in this study. **Table S2.** The antibodies for the Western blotting. **Table S3.** RNA-seq of differentially expressed genes in ATF3 overexpression SK-Hep1 cells. **Table S4.** RNA-seq of differentially expressed genes in ATF3 knockdown SMMC-7721 cells. **Table S5.** The antibodies for the IHC. **Figure S1.** The differential expression of ATF3 in HCC cell lines. **Figure S2.** ATF3 inhibited HCC cell proliferation in vitro by MTT assay. **Figure S3.** ATF3 suppressed HCC mobility in vitro. **Figure S4.** CYR61 suppressed HCC mobility in vitro. **Figure S5.** qRT-PCR analysis of the mRNA levels to confirm the differential expression of other genes identified from the ATF3 RNA-Seq results as targets of ATF3 in HCC cell lines with ATF3 overexpression or knockdown. (DOCX 3516 kb)

## Abbreviations

ATF3: Activating transcription factor 3
CCK-8: Cell counting kit-8
CYR61: Cysteine-rich angiogenic inducer 61
HCC: Hepatocellular carcinoma
MTT: 3-(4,5-dimethylthiazol-2-yl)-2,5-diphenyltetrazolium bromide
NC: Negative control
qRT-PCR: Quantitative real-time polymerase chain reaction
TFs: Transcription factors
TSS: Transcriptional start site
