# Supplementary material for: ATF3 inhibits the tumorigenesis and progression of hepatocellular carcinoma cells via upregulation of CYR61 expression
Source: J Exp Clin Cancer Res. 2018 Oct 30;37:263. doi: 10.1186/s13046-018-0919-8 (PMC6208028; doi:10.1186/s13046-018-0919-8)
Supplement: Supplementary file 1 — Table S1. Sequences of primers, probes and shRNA used for experiments in this study. Table S2. The antibodies for the Western blotting. Table S3. RNA-seq of differentially expressed genes in ATF3 overexpression SK-Hep1 cells. Table S4. RNA-seq of differentially expressed genes in ATF3 knockdown SMMC-7721 cells. Table S5. The antibodies for the IHC. Figure S1. The differential expression of ATF3 in HCC cell lines. Figure S2. ATF3 inhibited HCC cell proliferation in vitro by MTT assay. Figure S3. ATF3 suppressed HCC mobility in vitro. Figure S4. CYR61 suppressed HCC mobility in vitro. Figure S5. qRT-PCR analysis of the mRNA levels to confirm the differential expression of other genes identified from the ATF3 RNA-Seq results as targets of ATF3 in HCC cell lines with ATF3 overexpression or knockdown. (DOCX 3516 kb) [file 13046_2018_919_MOESM1_ESM.docx]

**Additional file 1**

**Title:** ATF3 inhibits the tumorigenesis and progression of hepatocellular carcinoma cells via upregulation of CYR61 expression

**Authors:**

Cong Chen^1^, Chao Ge^1^, Zheng Liu^2^, Liangyu Li^2^, Fangyu Zhao^1^, Hua Tian^1^, Taoyang Chen^3^, Hong Li^1^, Ming Yao^1^, Jinjun Li^1,*^

^1^State Key Laboratory of Oncogenes and Related Genes, Shanghai Cancer Institute, Renji Hospital, Shanghai Jiaotong University School of Medicine, Shanghai, China;

^2^Shanghai Medical College, Fudan University, Shanghai, China;

^3^Qidong Liver Cancer Institute, Qidong, China.

**Contents:**

**Supplementary Table 1-5**

**Supplementary Figure 1-5**

**Table S1 Sequences of primers, probes and shRNA used for experiments in this study**

| **Name** | **Primer Sequence (5’-3’)** |
| --- | --- |
| GAPDH-F | AGAAGGCTGGGGCTCATTTG |
| GAPDH-R | AGGGGCCATCCACAGTCTTC |
| ATF3-F | TTTGCTAACCTGACGCCCTT |
| ATF3-R | TGACTGATTCCAGCGCAGAG |
| CYR61-F | CGGCTCCCTGTTTTTGGAATG |
| CYR61-R | GGGTTTCTTTCACAAGGCGG |
| MMP28-F | GCCATCACTGTAGACAGGCAA |
| MMP28-R | TCTGAGACGTTGCCATCAGC |
| IL-11-F | GCGGACAGGGAAGGGTTAAAG |
| IL-11-R | AGGCGGCAAACACAGTTCA |
| HRH1-F | AGATGACACGGAGCTGTCGG |
| HRH1-R | CAGTTATGGCTCACTCCCTGGC |
| TMEM265-F | TTTGCCATCAAGGCGGAAGAG |
| TMEM265-R | GATGGCGTAGGAGAGCAACC |
| ATF3-Clone-F | CGGGATCCATGATGCTTCAACACCCAG |
| ATF3-Clone-R | CGGAATTCTTAGCTCTGCAATGTTCCT |
| shNC | TTCTCCGAACGTGTCACGT |
| shATF3-1 | CCGCCTTTCATCTGGATTCTA |
| shATF3-2 | GCTGAACTGAAGGCTCAGATT |
| shATF3-3 | CCTCTTTATCCAACAGATAAA |
| CYR61-Clone-F | CGGGATCCTTAGTCGTCACCCTTCTCCACTT |
| CYR61-Clone-R | CGGAATTCCATTGAACAGCCTGTAGAAGGG |
| shCYR61-1 | CTTCTACAGGCTGTTCAAT |
| shCYR61-2 | GAACCAGTCAGGTTTACTT |
| CYR61 (-818)-F | CGACGCGTGGAGACCTCTGCCTGGGAA |
| CYR61 (-437)-F | CGACGCGTGGCAAAGTTCTGAACTGGCC |
| CYR61 (+61)-F | CGACGCGTCCTCGAGCGAAAGACGC |
| CYR61 (+349)-F | CGACGCGTCCAGATTGCCCACGGCAGG |
| CYR61-Mut-F | CGACGCGTCCCCGCAGCTGTGTCGGGG |
| CYR61 (universal)-R | GAAGATCTGGAGCCTCTGATCTCGTCCA |
| CYR61-Ch-IP-F | CCAGATTGCCCACGGCAGG |
| CYR61-Ch-IP-R | GGAGCCTCTGATCTCGTCCA |

**Table S2 The antibodies for the Western blotting**

| **Antibody** | **Source** | **Catalogue number** | **Dilution** | **Application** | **Company** |
| --- | --- | --- | --- | --- | --- |
| **Primary antibodies for WB** | | | | | |
| ATF3 | Rabbit IgG | Ab200655 | 1:1000 | WB | Abcam |
| CYR61 | Rabbit IgG | #14479S | 1:1000 | WB | Cell Signaling |
| HRP-β-actin | Mouse | A3854 | 1:10000 | WB | Sigma-Aldrich |
| **Secondary antibodies for WB** | | | | | |
| HRP-anti-Rabbit IgG | Goat | A0545 | 1:3000 | WB | Sigma-Aldrich |

**Table S3 RNA-seq of differentially expressed genes in ATF3 overexpression SK-Hep1 cells**

| **Gene name** | **Description** | **Relative Fold Change** | **Q-value** |
| --- | --- | --- | --- |
| **Upregulated genes (ATF3/pWPXL)** | | | |
| CXCR4 | chemokine (C-X-C motif) receptor 4 | 8.035182498 | 0.006094249 |
| HRH1 | histamine receptor H1 | 6.195428704 | 1.75E-17 |
| EGR3 | early growth response 3 | 4.45466316 | 3.79E-06 |
| UPK1B | uroplakin 1B | 3.668562138 | 0.005171658 |
| IGFBP5 | insulin like growth factor binding protein 5 | 3.185779456 | 6.73E-09 |
| KLF10 | Kruppel-like factor 10 | 2.559640616 | 2.96E-08 |
| **CYR61** | **cysteine-rich, angiogenic inducer, 61** | **2.442292844** | **8.23E-08** |
| IL11 | interleukin 11 | 2.36510698 | 0.013410798 |
| FOXN3 | forkhead box N3 | 2.075580109 | 0.000620462 |
| **Downregulated genes (ATF3/pWPXL)** | | | |
| MMP28 | matrix metallopeptidase 28 | 0.228450772 | 3.87E-05 |
| TMEM265 | transmembrane protein 265 | 0.299480124 | 0.00085028 |
| SAMD9 | sterile alpha motif domain containing 9 | 0.381152295 | 9.38E-08 |
| CXCL8 | chemokine (C-X-C motif) ligand 8 | 0.40534663 | 0.000118526 |
| ID3 | inhibitor of DNA binding 3 | 0.409919139 | 7.90E-06 |
| ABCA1 | ATP binding cassette subfamily A member 1 | 0.42621923 | 2.46E-05 |
| SNAI2 | snail family zinc finger 2 | 0.444244884 | 1.24E-05 |
| KLF4 | Kruppel-like factor 4 | 0.447532468 | 0.000833128 |
| ID1 | inhibitor of DNA binding 1 | 0.466849637 | 3.87E-05 |

**Table S4 RNA-seq of differentially expressed genes in ATF3 knockdown SMMC-7721 cells**

| **Gene name** | **Description** | **Relative Fold Change** | **Q-value** |
| --- | --- | --- | --- |
| **Upregulated genes (shATF3/shNC)** | | | |
| CLDN2 | claudin 2 | 4.518028364 | 0.000786281 |
| FGFBP1 | fibroblast growth factor binding protein 1 | 3.664147852 | 3.34E-05 |
| TMEM265 | transmembrane protein 265 | 3.574975571 | 0.049003639 |
| MAPK4 | mitogen-activated protein kinase 4 | 2.660608129 | 2.56E-07 |
| S100A4 | S100 calcium binding protein A4 | 2.540864724 | 9.92E-09 |
| ABCB1 | ATP binding cassette subfamily B member 1 | 2.51992959 | 6.11E-05 |
| MMP19 | matrix metallopeptidase 19 | 2.311145886 | 0.008308255 |
| CCNE2 | cyclin E2 | 2.195191115 | 8.77E-05 |
| PSCA | prostate stem cell antigen | 2.166162975 | 7.02E-05 |
| MMP28 | matrix metallopeptidase 28 | 2.03084128 | 0.007035726 |
| INSIG1 | insulin induced gene 1 | 2.024338098 | 4.26E-05 |
| **Downregulated genes (shATF3/shNC)** | | | |
| GDF15 | growth differentiation factor 15 | 0.076437984 | 3.63E-45 |
| HRH1 | histamine receptor H1 | 0.331935163 | 0.000217605 |
| IL11 | interleukin 11 | 0.34122141 | 1.05E-05 |
| KLF6 | Kruppel-like factor 6 | 0.388888723 | 9.15E-07 |
| E2F3 | E2F transcription factor 3 | 0.426329169 | 2.34E-05 |
| **CYR61** | **cysteine-rich, angiogenic inducer, 61** | **0.445304987** | **4.15E-05** |
| FN1 | fibronectin 1 | 0.455231545 | 0.00189451 |
| SCAI | suppressor of cancer cell invasion | 0.463820808 | 0.001032097 |
| CAV2 | caveolin 2 | 0.483784984 | 0.000465265 |
| IGFBP7 | insulin like growth factor binding protein 7 | 0.498065 | 0.000549101 |

**Table S5 The antibodies for the IHC**

| **Antibodies** | **Source** | **Catalogue number** | | **Dilution** | **Application** | **Company** |
| --- | --- | --- | --- | --- | --- | --- |
| **Primary antibodies for IHC** | | | | | | |
| ATF3 | Mouse IgG | | Ab191513 | 1:25 | IHC | Abcam |
| CYR61 | Rabbit IgG | | 26689-1-AP | 1:100 | IHC | Proteintech |
| **Secondary antibody for IHC** | | | | | | |
| EnViSion | Detection | | Kit K5007 | --- | IHC | DAKO |
| (Peroxidase/DAB; Rabbit/Mouse) | | | | | | |

**
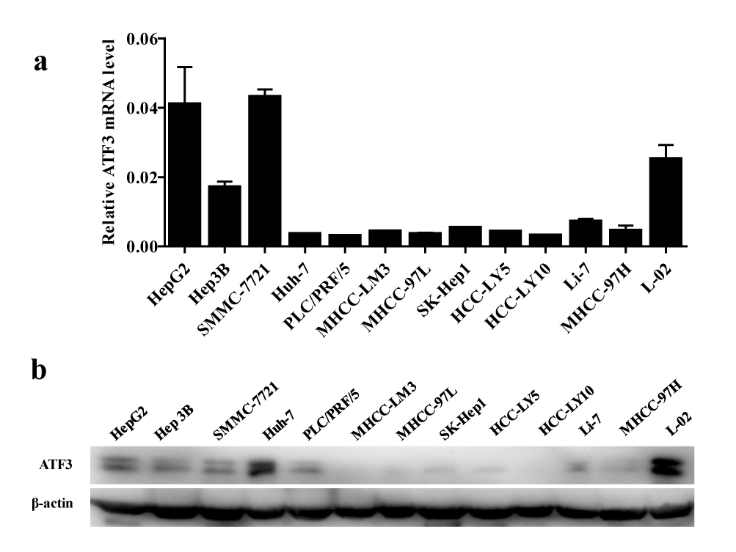
**

**Figure S1. The differential expression of ATF3 in HCC cell lines.** The expression levels of ATF3 mRNA (**a**) and protein (**b**) were respectively detected by qRT-PCR and western blot in HCC cell lines as well as in the immortalized normal hepatocyte cell line L-02. Data are presented as the means ± S.D. from experiments with three replicates. β-Actin was used as a loading control.


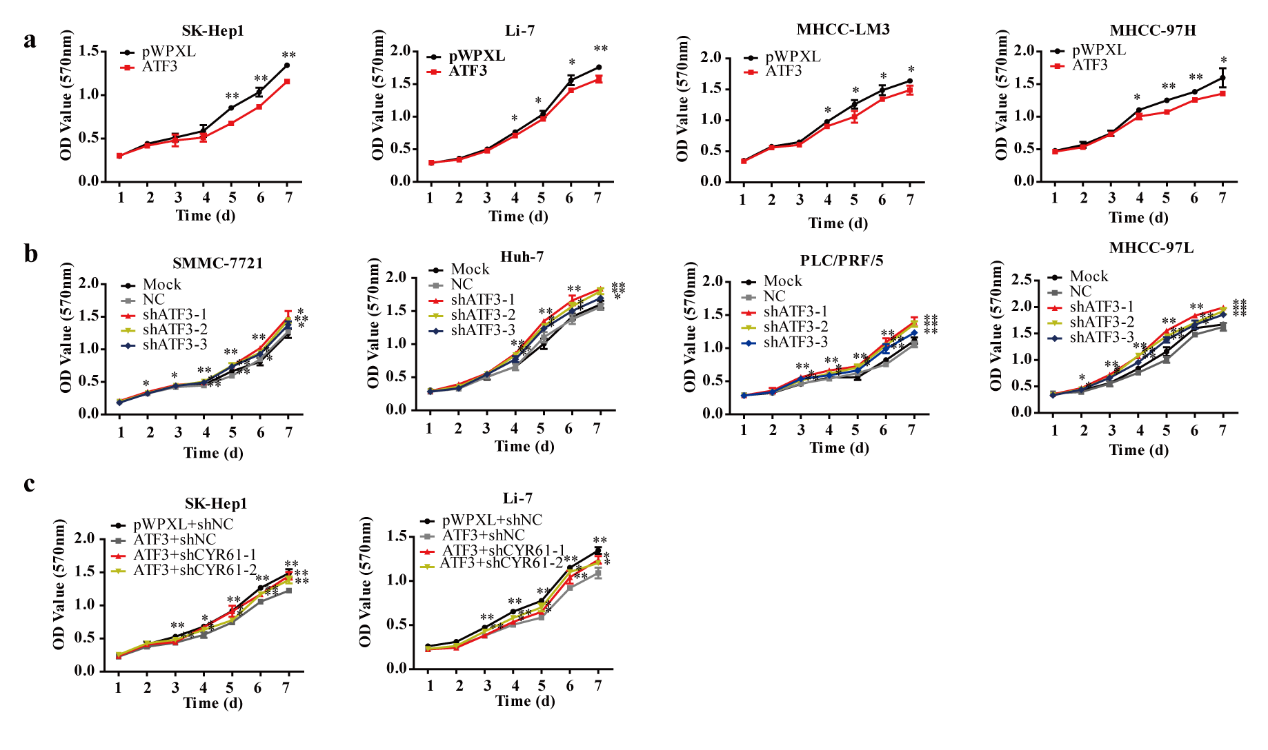


Figure S2. **ATF3 inhibited HCC cell proliferation *in vitro* by MTT assay.** (a) The cell proliferative ability of HCC cells with ATF3 overexpression was inhibited; **(b)** The cell proliferative ability of HCC cells with ATF3 knockdown was increased; **(c)** Knockdown CYR61 in HCC cells overexpressing ATF3 rescued the suppressive proliferative ability of ATF3 as indicated by the MTT assay. The bar graphs in (**a**), (**b**) and (**c**) represent quantitative data from three replicates. Unpaired Student’s *t*-test was used for statistical analysis, and the data are shown as the mean ± S.D. **P* < 0.05, ***P* < 0.01 and ns: no significance.

**
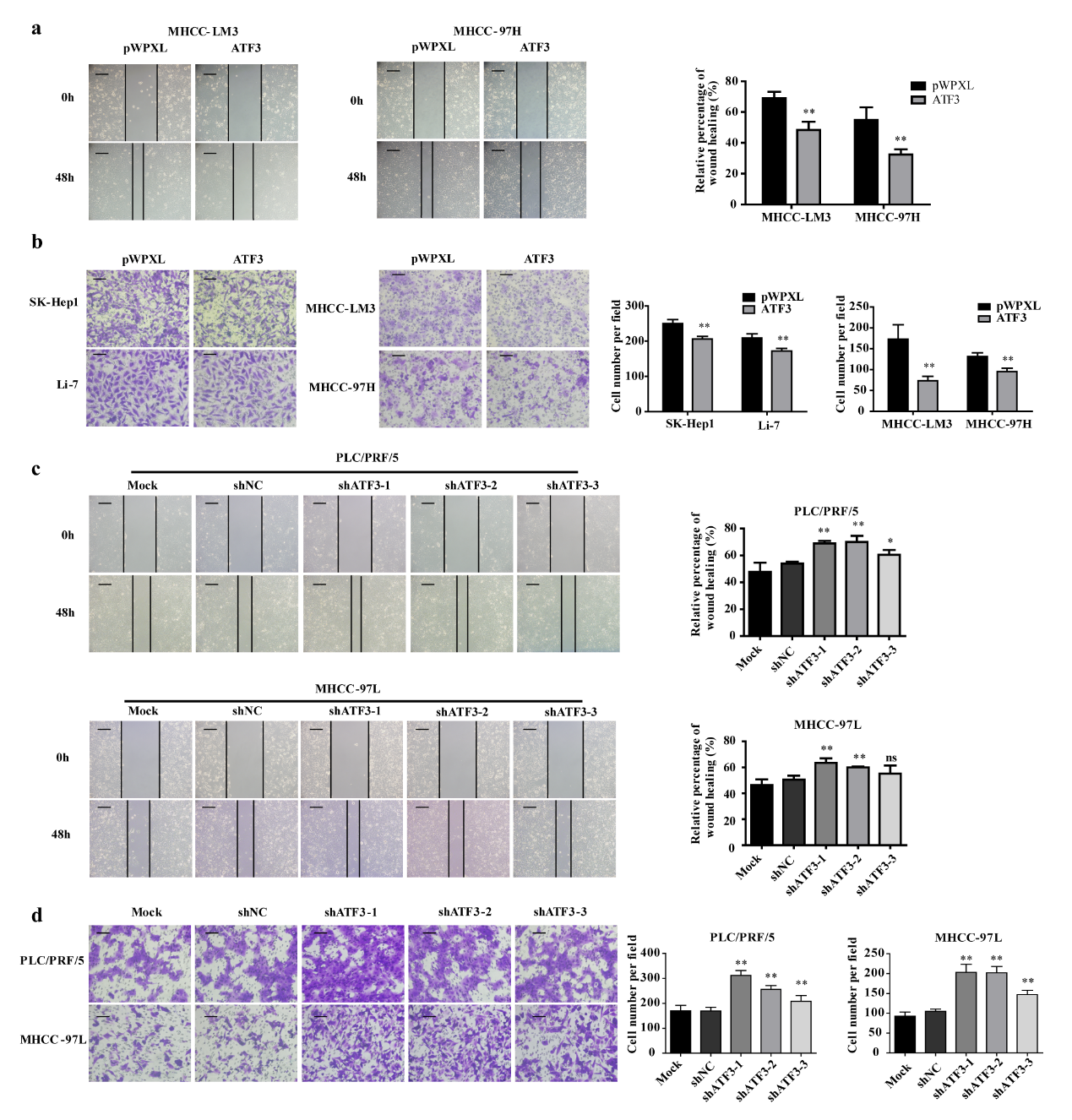
**

**Figure S3. ATF3 suppressed HCC mobility *in vitro.*** (**a**) MHCC-LM3 and MHCC-97H cells overexpressing ATF3 showed suppressed HCC cell migration *in vitro* as evaluated by the wound healing assay (scale bar, 200 μm). (**b**) Overexpression of ATF3 suppressed HCC cell invasion *in vitro* as evaluated by the transwell assay (scale bar, 100 μm). ATF3 knockdown in PLC/PRF/5 and MHCC-97L cells promoted HCC cell migration (**c**) and invasion (**d**) *in vitro* as shown by the wound healing assay (scale bar, 200 μm) and transwell assay (scale bar, 100 μm), respectively. The bar graphs in (**a**), (**b**), (**c**) and (**d**) represent quantitative data from three replicates. Unpaired Student’s *t*-test was used for statistical analysis, and the data are shown as the mean ± S.D. **P* < 0.05, ***P* < 0.01 and ns: no significance.


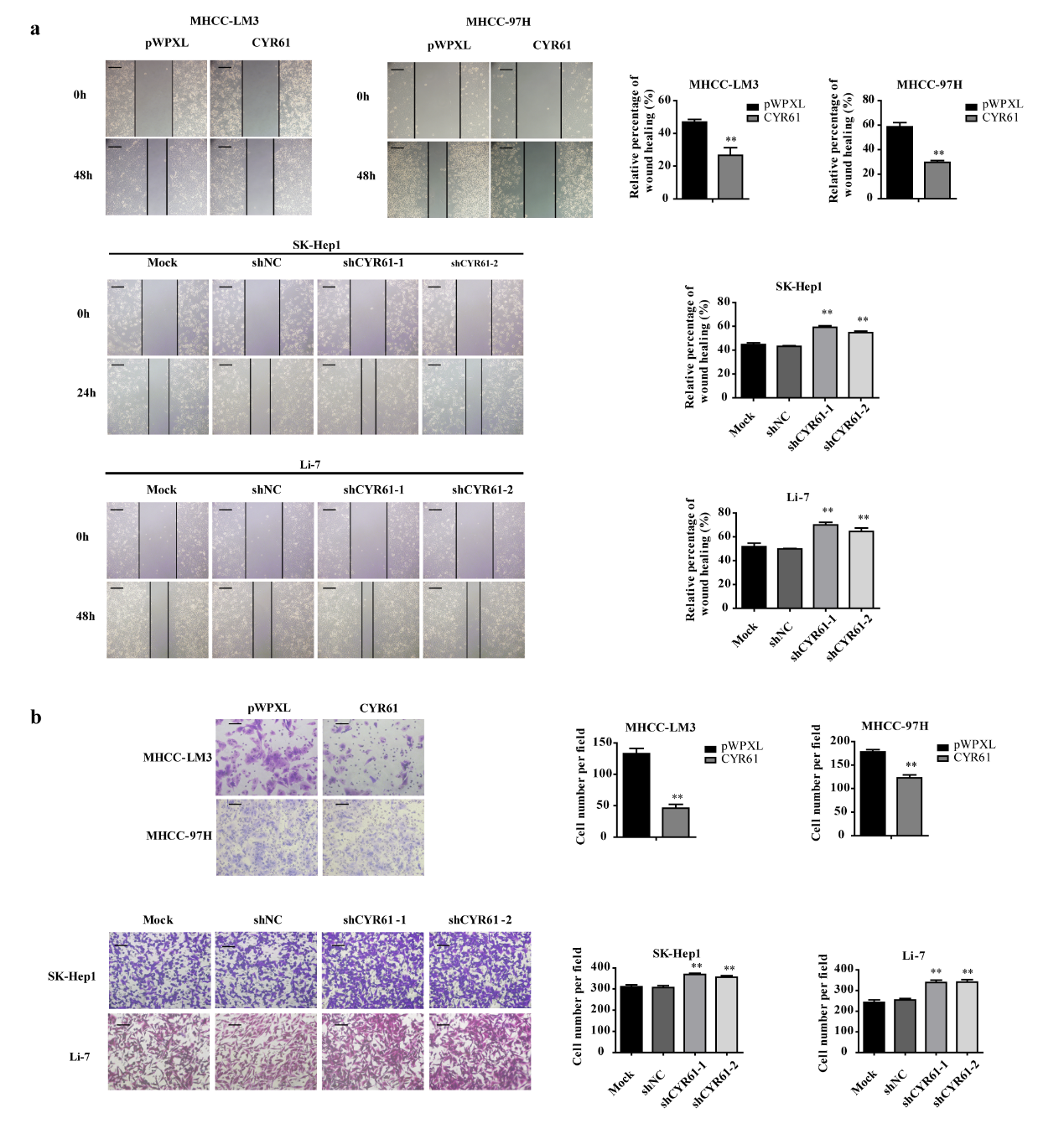


**Figure S4. CYR61 suppressed HCC mobility *in vitro.*** (**a**) MHCC-LM3 and MHCC-97H cells overexpressing CYR61 showed suppressed HCC cell migration and SK-Hep1 and Li-7 cells silencing CYR61 showed increased HCC cell migration *in vitro* as evaluated by the wound healing assay (scale bar, 200 μm). (**b**) Overexpression of CYR61 in MHCC-LM3 and MHCC-97H cells suppressed HCC cell invasion, knockdown of CYR61 in SK-Hep1 and Li-7 cells promoted HCC cell invasion *in vitro* as evaluated by the transwell assay (scale bar, 100 μm). The bar graphs in (**a**) and (**b**) represent quantitative data from three replicates. Unpaired Student’s *t*-test was used for statistical analysis, and the data are shown as the mean ± S.D. **P* < 0.05, ***P* < 0.01 and ns: no significance.


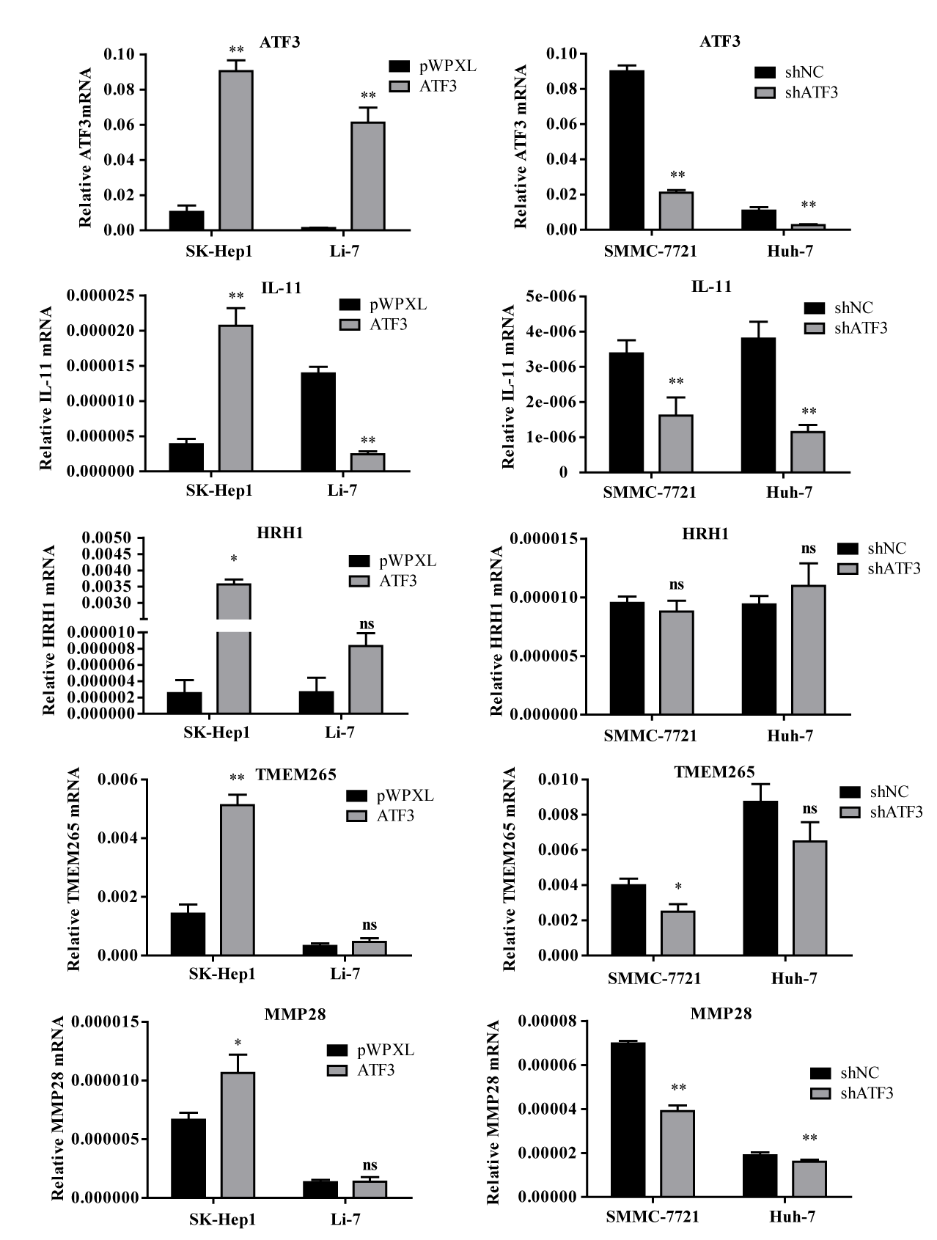


**Figure S5.** **qRT-PCR analysis of the mRNA levels to confirm the differential expression of other genes identified from the ATF3 RNA-Seq results as targets of ATF3 in HCC cell lines with ATF3 overexpression or knockdown**. The bar graph represents quantitative data from three independent experiments. Data are shown as the mean **±** S.D. from experiments with three replicates. **P* < 0.05, ***P* < 0.01 and ns: no significance.
